# Is time to first CT scan in patients with isolated severe traumatic brain injury prolonged when prehospital arterial cannulation is performed? A retrospective non-inferiority study

**DOI:** 10.1186/s13049-024-01251-9

**Published:** 2024-09-05

**Authors:** Michael Eichlseder, Sebastian Labenbacher, Alexander Pichler, Michael Eichinger, Thomas Kuenzer, Philipp Zoidl, Barbara Hallmann, Felix Stelzl, Nikolaus Schreiber, Paul Zajic

**Affiliations:** 1 Division of Anaesthesiology and Intensive Care Medicine 1, Medical University of Graz, Auenbruggerplatz 5, 8036 Graz, Austria; 2 Institute for Medical Informatics, Statistics and Documentation, Medical University of Graz, Graz, Austria; 3 Medical University of Graz, Graz, Austria; 4 Division of Anaesthesiology and Intensive Care Medicine 2, Medical University of Graz, Graz, Austria

**Keywords:** Brain Injuries, Traumatic, Emergency medical services, Blood pressure, Arterial pressure, Hemodynamic monitoring, Anaesthesia, Intubation

## Abstract

**Background:**

Invasive blood pressure measurement is the in-hospital gold standard to guide hemodynamic management and consecutively cerebral perfusion pressure in patients with traumatic brain injury (TBI). Its prehospital use is controversial since it may delay further care. The primary aim of this study was to test the hypothesis that patients with severe traumatic brain injury who receive prehospital arterial cannulation, compared to those with in-hospital cannulation, do not have a prolonged time between on-scene arrival and first computed tomography (CT) of the head by more than ten minutes.

**Methods:**

This retrospective study included patients 18 years and older with isolated severe TBI and prehospital induction of emergency anaesthesia who received treatment in the resuscitation room of the University Hospital of Graz between January 1st, 2015, and December 31st, 2022. A Wilcoxon rank-sum test was used to test for non-inferiority (margin = ten minutes) of the time interval between on-scene arrival and first head CT.

**Results:**

We included data of 181 patients in the final analysis. Prehospital arterial line insertion was performed in 87 patients (48%). Median (25–75th percentile) durations between on-scene arrival and first head CT were 73 (61–92) min for prehospital arterial cannulation and 75 (60–93) min for arterial cannulation in the resuscitation room. Prehospital arterial line insertion was significantly non-inferior within a margin of ten minutes with a median difference of 1 min (95% CI − 6 to 7, *p* = 0.003).

**Conclusion:**

Time-interval between on-scene arrival and first head CT in patients with isolated severe traumatic brain injury who received prehospital arterial cannulation was not prolonged compared to those with in-hospital cannulation. This supports early out-of-hospital arterial cannulation performed by experienced providers.

## Background

Traumatic brain injury (TBI) is the most common injury leading to death and disability in the western world [1]. The estimated incidence of TBI from 2009 to 2011 was around 300 per 100,000 inhabitants per year, accounting for approximately 2000 cases of severe TBI every year in Austria [2, 3].

The initial prehospital management of patients with severe TBI includes minimising secondary brain damage as well as rapid transportation to a centre with specific diagnostic and therapeutic possibilities [4, 5].

To minimise secondary damage and enable an adequate cerebral perfusion pressure, stable hemodynamic conditions as well as adequate oxygenation and decarboxylation are required. The latter two aspects can often only be achieved with prehospital anaesthesia to protect the airway and allow controlled ventilation, especially in cases of severe TBI [5].

However, TBI is often accompanied by hemodynamic compromise which can be even further exacerbated by induction of anaesthesia and positive pressure ventilation [6]. To detect potential hemodynamic changes and enable rapid interventions, arterial cannulation and continuous real-time blood pressure monitoring has become the in-hospital gold standard [7].

While holding the potential for benefits, arterial cannulation and invasive blood pressure measurement are techniques rarely used in the prehospital setting. Apart from technical skills required, the duration spent on scene could also be prolonged. Therefore, all contributing elements demand a comprehensive evaluation. Due to challenging conditions, prehospital cannulation and setup of the invasive blood pressure monitoring could require more time outside the hospital. This could potentially delay the first major and decisive diagnostic step, a computed tomography (CT) of the head. The Austrian working group for improvement of early TBI care suggests a head CT within twenty to thirty minutes after hospital admission, giving a range of ten minutes [8].

Data comparing those time spans is still lacking and has been called for in a recent publication [9]. This was published by a helicopter emergency medical service (HEMS) based in the United Kingdom, offering a retrospective observational review of practice of over one thousand prehospital arterial cannulations in a mixed population, mostly cardiac arrest [9].

The primary aim of this study was to test the hypothesis that patients with severe TBI who receive prehospital arterial cannulation, compared to those with in-hospital cannulation, do not have a prolonged time-interval between on-scene arrival and first head CT by more than ten minutes.

## Methods

### Study design and setting

This study was a single-centre, retrospective analysis of routine data conducted according to the Strengthening the Reporting of Observational Studies in Epidemiology (STROBE) guideline [10]. It is based on the principles of positivism and guided by an analytical approach.

Data from the local trauma registry, which includes all major trauma patients treated in the resuscitation room, was used to identify eligible patients and extract the relevant data from the resuscitation room treatment.

The University Hospital of Graz is a major trauma centre with a vast catchment area covering approximately 1.5 million individuals. Both physician staffed ground-based response units as well as physician staffed HEMS transfer severely injured patients to this centre. Prehospital emergency physicians undergo specific prehospital training in addition to their background specialty (mostly anaesthesia, internal medicine or intensive care medicine) and are paired with a paramedic. These specialized units are dispatched to severe traumatic, neurologic, pediatric, medical, and toxicological emergencies as well as cardiopulmonary resuscitations and carry equipment for arterial cannulation and invasive blood pressure measurement, which can be performed at the physician’s discretion [11].

### Selection of participants

Data of missions between 1st January, 2015, and 31st December, 2022, was extracted. Patients 18 years and older with an isolated severe TBI (defined as abbreviated injury scale (AIS) ≥ 3 in the body region “head”, an initial Glasgow Coma Scale (GCS) < 9, and no AIS > 2 in any other body region than “head”) who received prehospital induction of emergency anaesthesia and airway management were eligible for this study [12, 13].

Patients were excluded if there was no head CT directly after initial treatment in the resuscitation room, if no arterial line insertion prior to head CT was performed, or if data regarding the primary outcome was missing.

### Measurements

The time point (dichotomous, either prehospital or in-hospital) of arterial line insertion was extracted from the prehospital record or the resuscitation room record. Patient and case-specific data (age, gender, type of injury, outcome), relevant timepoints (time of on-scene arrival (at the patient), time of transport start, handover time, time of first head CT), admission blood pressure, initial GCS, vasopressor requirement, and transport modality were extracted from the source data of the local trauma registry. Any still missing data were extracted from the prehospital record or the resuscitation room record.

### Outcomes

Primary outcome was the time-interval between on-scene arrival of the prehospital physician and first head CT. Secondary endpoints were time between on-scene arrival of the prehospital physician and handover in the resuscitation room, time on-scene of the prehospital physician, time between handover in the resuscitation room and first head CT, and the rate of patients who were hypotensive on arrival in the resuscitation room (defined as a) systolic blood pressure below 90 mmHg and b) below 110 mmHg in patients 18 to 49 years old as well as 70 years old and older and below 100 mmHg in patients 50–69 years old according to the 4th brain trauma foundation guideline) [4].

### Analysis

Sample size calculation was based on the assumption that 40% of the patients receive prehospital arterial cannulation, as per internal data from one ground-based response unit. Further, the duration from on-scene arrival until the first head CT was assumed to be 87.7 min (SD ± 33.4 min), based on a prior publication [14]. With a non-inferiority margin of ten minutes based on recommendations for the initial management of TBI by the Austrian TBI improvement working group and a one-sided significance level of 2.5%, 380 patients were required to achieve a power of 80% [8]. Assuming 50 patients with isolated severe TBI per year, the study period of 8 years (2015–2022) was chosen. Sample size calculation was performed using nQuery (Dotmatics, Boston, MA, USA).

Demographic, injury-related and treatment-related data were presented as mean and standard deviation (SD), median and 25–75th percentile, or number (*n*) and percentages (%), as appropriate.

To evaluate the primary endpoint, a Wilcoxon rank-sum test was used to test for non-inferiority at a non-inferiority margin of ten minutes. A *p*-value below 0.025 was considered statistically significant.

To evaluate the secondary endpoints (time between on-scene arrival and handover, time on-scene, time from handover to first head CT, patients hypotensive at resuscitation room handover), continuous variables were compared using the Wilcoxon rank-sum test for continuous variables and Fisher’s exact test for discrete. Two-sided *p*-values below 0.05 and one-sided *p*-values below 0.025 were considered significant.

Although not pre-specified, we conducted a subgroup analysis with respect to mode of transport due to the higher percentage of patients transported by HEMS in the prehospital group. In addition to the descriptive analysis, multiple linear regression with an interaction term between mode of transport and prehospital arterial cannulation was used to test if duration between on-scene arrival and first head CT was significantly affected by the mode of transport and prehospital arterial cannulation.

All analyses were performed using R version 4.3 (R Core Team (2023). R: A Language and Environment for Statistical Computing. R Foundation for Statistical Computing, Vienna, Austria. https://www.R-project.org/), graphs were created using R version 4.3 and Microsoft Excel 2016 (Microsoft Corp, Redmond, WA, USA).

## Results

**Fig. 1:**
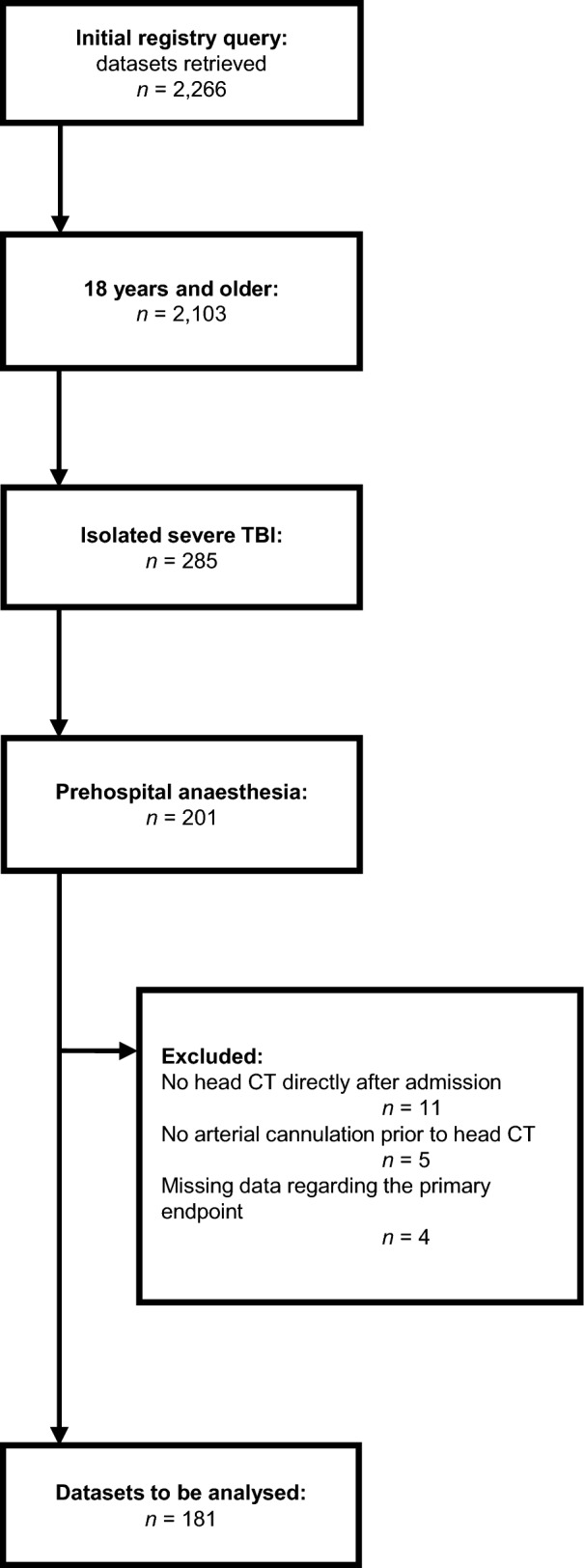
Study flow chart. TBI = traumatic brain injury, CT = computed tomography

Out of a total of 2,266 patients documented in the local trauma registry during the selected timespan, 201 patients with an age of 18 years or older had an isolated severe TBI according to the above outlined definition and received prehospital emergency anaesthesia. After exclusion of eleven patients without an immediate head CT, five patients without arterial cannulation prior to head CT and four patients with missing data regarding the primary endpoint, 181 could be included in the final analysis, falling short of the calculated target sample size. The exact selection process is depicted in Fig. 1. Of these 181 patients, 87 had prehospital arterial cannulation (prehospital group), and 94 had in-hospital arterial cannulation (in-hospital group). In the prehospital group, median (25–75th percentile) age was 66 (49–79) years with 70% (n = 61) males compared to a median age of 66 (52–77) years and 64% (n = 60) males in the in-hospital group. Further baseline patient characteristics and comparisons between the two groups are shown in Table 1.

**Table 1:** Characteristics of the study population (n = 181)

Characteristic	Prehospital cannulation	In-hospital cannulation
n	87	94
Age [years]—median (25–75th percentile)	66 (49–79)	66 (52–77)
Male sex—n (%)	61 (70.1)	60 (63.8)
Mechanism of injury—n (%)		
Violence	8 (9.2)	9 (9.6)
Fall < 3 m	54 (62.1)	54 (57.4)
Fall > 3 m	6 (6.9)	8 (8.4)
Traffic	16 (18.4)	20 (21.3)
Other	3 (3.4)	3 (3.2)
Injury Severity Score—median (25–75th percentile)	25 (20–26)	25 (21–29)
Abbreviated Injury Scale Head—n (%)		
3	10 (11.5)	7 (7.4)
4	22 (25.3)	20 (21.3)
5	55 (63.2)	66 (70.2)
6	0 (0)	1 (1.1)
Initial Glasgow Coma Scale—median (25–75th percentile)	4 (3–6)	4 (3–6)
Mode of Transport—n (%)		
Ground based	28 (32.9)	42 (45.2)
HEMS	57 (67.1)	51 (54.8)
Prehospital vasopressor—n (%)		
Applied	26 (34.2)	27 (30.7)
Prehospital fluid volume [ml]—median (25–75th percentile)	500 (500–1000)	500 (500–1000)
28-day mortality—n (%)	36 (41.4)	47 (50)

*m* meter, *HEMS* helicopter emergency medical service, *ml* milliliter

### Primary outcome

**Fig. 2:**
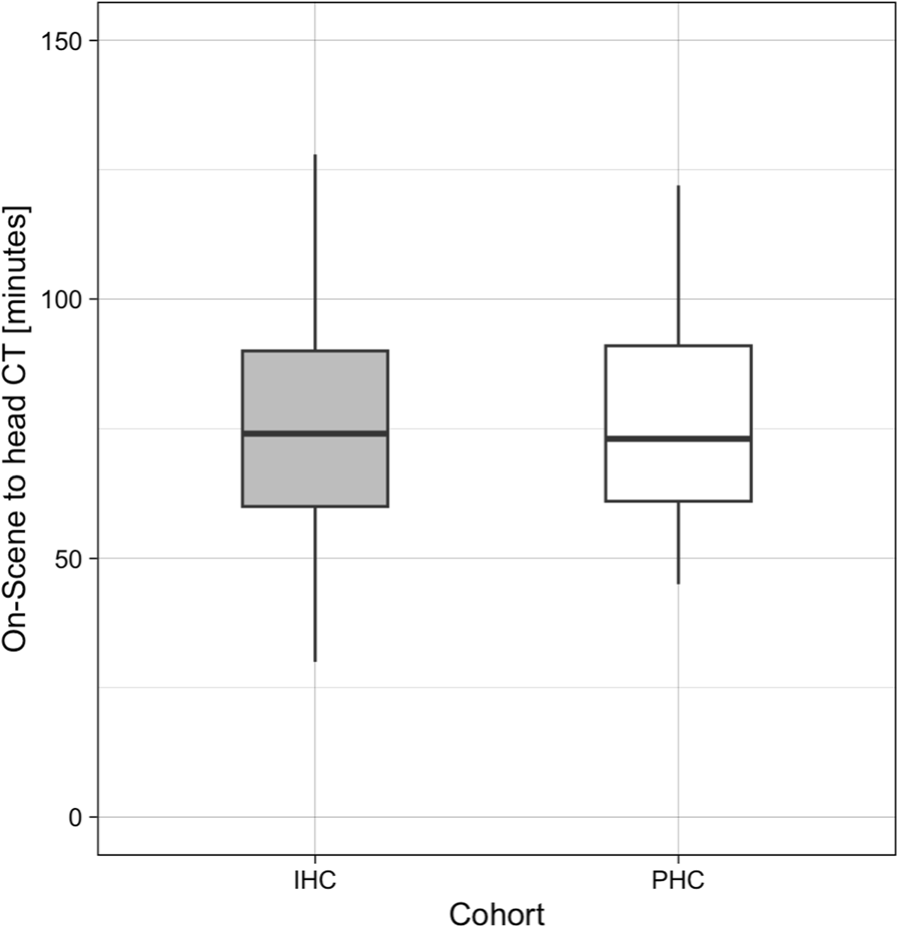
Boxplot comparison of the time-interval between on-scene arrival and the first head CT after hospital admission between the in-hospital and prehospital arterial cannulation groups. CT = computed tomography, IHC = in-hospital cannulation, PHC = prehospital cannulation

Median durations between on-scene arrival and first head CT were 73 (61–92) minutes for prehospital arterial cannulation and 75 (60–93) minutes for arterial cannulation in the resuscitation room, as shown in Fig. 2. Prehospital arterial line insertion was significantly non-inferior within a margin of ten minutes with a median difference of 1 min (95% CI − 6 to 7, *p* = 0.003).

### Secondary outcomes

**Fig. 3:**
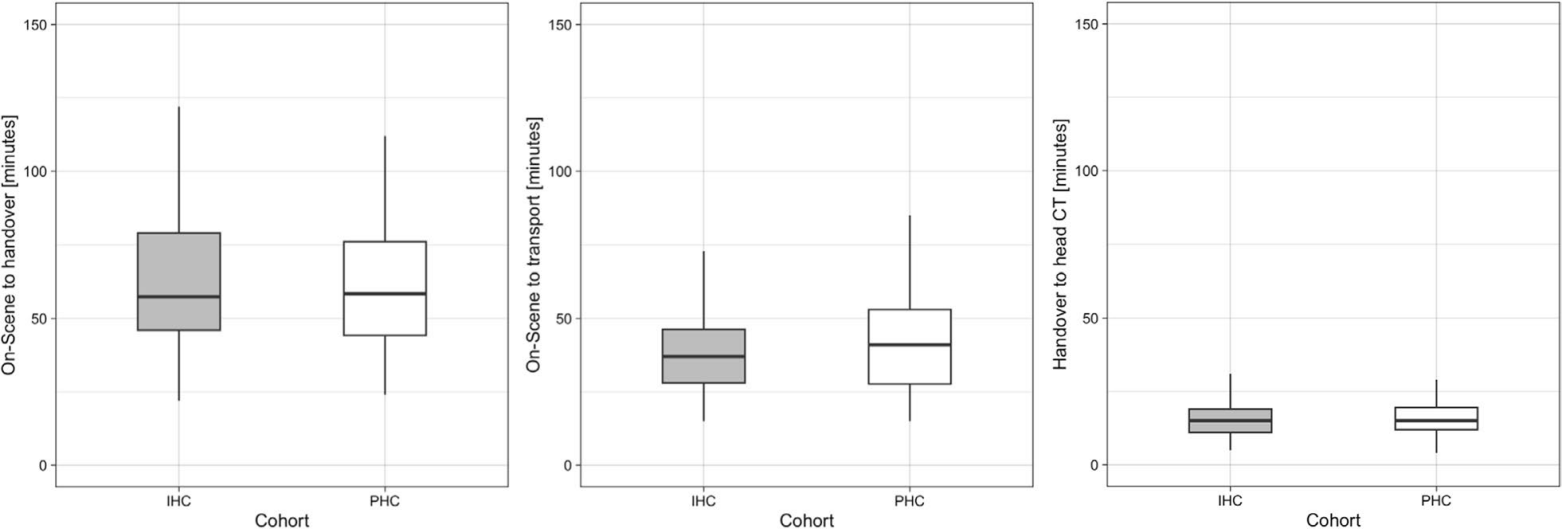
Boxplot comparisons of the time-interval between (**A**) on-scene arrival and hospital handover, (**B**) on-scene arrival and transport, and (**C**) hospital handover and first head CT after hospital admission between the in-hospital and prehospital arterial cannulation groups. CT = computed tomography, IHC = in-hospital cannulation, PHC = prehospital cannulation

No significant differences were found regarding time between on-scene arrival and handover in the resuscitation room (median difference 0 min, 95% CI (− 7, 7)), time on scene (median difference 3 min, 95% CI (− 2, 7)), and time between handover in the resuscitation room and first head CT (median difference 0 min, 95% CI (− 2, 2)). Further details regarding the selected time intervals are shown in Table 2 and Fig. 3.

**Table 2:** Median duration in minutes of the selected time-intervals in the overall, prehospital cannulation and in-hospital cannulation cohort

Characteristic	Overall	Prehospital cannulation	In-hospital cannulation
n	181	87	94
On-Scene to head CT [minutes]—median (25–75th percentile)	74 (61–93)	73 (61–92)	75 (60–93)
On-Scene to handover [minutes]—median (25–75th percentile)	58 (45–77)	58 (44–76)	57 (46–79)
On-Scene to transport [minutes]—median (25–75th percentile)	38 (28–49)	41 (28–53)	37 (28–46)
Handover to head CT [minutes]—median (25–75th percentile)	15 (11–19)	15 (12–20)	15 (11–19)

*CT* computed tomography

Median blood pressure at handover in the resuscitation room was 128 (106–150) mmHg systolic and 80 (60–84) mmHg diastolic in the prehospital group compared to 130 (110–147.25) mmHg systolic and 80 (62–95.25) mmHg diastolic in the in-hospital group. No significant differences in the rates of admission blood pressure below 90 mmHg systolic (prehospital group: 8 out of 79 (10%); in-hospital group: 9 out of 90 (10%), *p* = 0.98) nor according to the recommended blood pressure targets according to the 4th TBI guideline (prehospital group: 20 out of 79 (25%); in-hospital group: 19 out of 90 (21%), *p* = 0.52) were found.

### Exploratory subgroup analysis

Data was divided into two subgroups according to mode of transportation. In the HEMS subgroup, the median duration from on-scene arrival to the first head CT was 71 (61–93) minutes and on-scene time was 38 (26–54) minutes in the prehospital group. In the in-hospital group, a median of 67 (58–82) minutes passed from on-scene arrival to the head CT and a median of 34 (25–43) minutes were spent on scene. For the primary outcome duration from on-scene arrival to the first head CT, the least-square means of the group differences between prehospital versus in-hospital are 6.1 min (95% CI: − 5.3 to 17.5) in the HEMS subgroup and -6.2 min (95% CI − 20.6 to 8.2) in the subgroup of ground-based transportation, with a *p*-value of 0.19 for interaction resulting non-significant.

## Discussion

In this retrospective study, the duration between on-scene physician arrival and first head CT was almost identical in patients with prehospital arterial cannulation and patients with arterial cannulation in the resuscitation room (73 versus 75 min) and prehospital arterial line insertion was significantly non-inferior.

This can be attributed to several potential factors. In the study’s specific region, physicians specialising in prehospital emergency care predominantly possess backgrounds in anaesthesiology or intensive care medicine where arterial cannulation is a routine practice.

Additionally, the observation of prehospital arterial cannulation in nearly every second patient underlines its sustained and frequent utilisation as a standard intervention in these prehospital services. This regular exposure ensures that teams as a whole can perform the procedure of arterial cannulation and invasive blood pressure monitoring in a timely fashion. This is further supported by the noteworthy fact that the median time between on-scene arrival and head CT in this study is 74 min in contrast to the average of 87.7 min described in the German Trauma Registry [14].

Median on-scene times was 41 (28–53) min and 37 (28–46) min in the prehospital and in-hospital cohort, respectively. This aligns with earlier data from *Wildner *et al*.* investigating the feasibility of out-of-hospital arterial cannulation [11]. In their prospective observational study, prehospital arterial cannulation in a mixed patient population required a median of two minutes and setup of the invasive blood pressure monitoring three minutes. Time between on-scene arrival and handover in the resuscitation room was almost alike (58 versus 57 min) in the two groups and similar to previously published data from other prehospital physician staffed services in Europe. For example, median total mission time was 69 (53–92) min in a study investigating severe TBI from Finland and 66 (51–80) min in a Dutch TBI cohort receiving prehospital induction of emergency anaesthesia by a physician staffed HEMS service [15, 16]. These data show, that arterial cannulation in the out of hospital setting does not prolong treatment times in a, to our opinion, clinically relevant fashion.

No difference between the two groups regarding the admission blood pressure was found. This is in contrast to in-hospital data where invasive blood pressure monitoring enhanced the detection of hypotension and also reduced hypotension during the anaesthesia induction process significantly, which also led to the recommendation to establish invasive blood pressure prior to anaesthetic induction in high risk in-hospital patients [17–19]. This discrepancy could be attributed to the methodology employed in our study, wherein only the blood pressure at a single time point (handover in the resuscitation room) could be evaluated. A state of hemodynamic equilibrium is frequently attained by the time of patient handover, as critical interventions with a high risk of hypotension, for instance induction of anaesthesia, have already been performed on-scene. Therefore, the out-of-hospital hemodynamic parameters would be of great interest. However, the prehospital blood pressures could not be compared as the handwritten prehospital records do not provide the necessary granularity and accuracy of vital parameters to use them for scientific purposes. To thoroughly assess the potential impact on blood pressure and 28-day mortality—which was 41% in the prehospital group compared to 50% in the in-hospital group—a prospective trial appears necessary.

Despite similar injury and baseline characteristics, a higher percentage of patients who received prehospital arterial line placement were treated by HEMS. An explanation for this phenomenon could be that HEMS doctors, often experienced prehospital physicians, may exhibit more familiarity and routine in prehospital arterial cannulation, thus maintaining a lower threshold for its utilisation. To further evaluate this, an unplanned subgroup analysis was performed. It revealed similar time intervals compared to the overall cohort and the time from on-scene arrival to head CT as well as on-scene time between the prehospital and in-hospital group were almost identical in the HEMS subgroup. Furthermore, multiple linear regression indicated no significant interaction of prehospital arterial cannulation with the mode of transport in explaining the duration from on-scene arrival to the first head CT.

### Limitations

There are several limitations to this study. The planned sample size of 380 was not met as fewer isolated severe traumatic brain injuries than anticipated occurred in the selected timespan. Although the initially planned sample size was not reached, the inclusion period was not prolonged due to sufficient data for answering the research question with highly significant results. Furthermore, with potential practice changes over time and a decrease in data quality, a prolonged inclusion period would also increase the risk of bias. It is a retrospective study and patients were therefore not randomised. Still, both groups had a similar injury severity as shown in Table 1. A major limitation arises from the fact that only patients with successful arterial cannulation could be included. With a previously published success rate of approximately 84% at the local prehospital physician response unit, this fraction of patients should, however, only be small [11]. Additionally, due to its retrospective design, the data is confined to its originally documented content. Nevertheless, the use of a standardised resuscitation room record, data re-evaluation by two members of the study team, and comparison of time points with those documented by the emergency medical dispatch centre contribute to the overall quality of the data.

## Conclusion

In summary, prehospital arterial cannulation appears to be non-inferior compared to cannulation in the resuscitation room regarding time between on-scene arrival and first head CT in patients with isolated severe TBI, although further research is needed to confirm. This supports the early, out-of-hospital arterial cannulation performed by an experienced provider.

## Data Availability

The datasets used and/or analysed during the current study are available from the corresponding author on reasonable request.

## Abbreviations

AIS: Abbreviated injury scale
CT: Computed tomography
CI: Confidence interval
GCS: Glasgow coma scale
HEMS: Helicopter emergency medical service
SD: Standard deviation
TBI: Traumatic brain injury
